# Value of Multimodal Imaging Approach to Diagnosis of Neurosarcoidosis

**DOI:** 10.3390/brainsci9100243

**Published:** 2019-09-21

**Authors:** Ilaria Sammarra, Gaetano Barbagallo, Angelo Labate, Baldassare Mondello, Giuseppe Albonico, Maurizio Maisano, Giuseppe Lucio Cascini, Aldo Quattrone, Antonio Gambardella

**Affiliations:** 1 Institute of Neurology, Magna Græcia University, 88100 Catanzaro, Italy; ilariasammarra@gmail.com (I.S.); barbagallo@unicz.it (G.B.); a.gambardella@unicz.it (A.G.); 2 Unit of Thoracic Surgery, Grande Ospedale Metropolitano di Reggio Calabria, 89124 Reggio Calabria, Italy; baldassare.mondello@gomrc.it; 3 Unit of Anatomical Pathology, Grande Ospedale Metropolitano di Reggio Calabria, 89124 Reggio Calabria, Italy; giuseppe.albonico@gomrc.it (G.A.); maruzio.maisano@gomrc.it (M.M.); 4 Institute of Radiology, Nuclear Medicine Unit, Magna Graecia University, 88100 Catanzaro, Italy; cascini@unicz.it; 5 Neuroimaging Research Unit, IBFM, National Research Council, 88100 Catanzaro, Italy; quattrone@unicz.it; 6 Neuroscience Research Centre, Magna Græcia University, 88100 Catanzaro, Italy

**Keywords:** neurosarcoidosis, multimodal imaging, magnetic resonance imaging, computed tomography, magnetic resonance spectroscopy, positron emission tomography

## Abstract

Background: Neurosarcoidosis is a highly variable condition with many clinical and radiological manifestations, that can lead to difficult identification of isolated central nervous system (CNS) forms, because it could mimic inflammatory, infective or neoplastic disorders. Conventional magnetic resonance imaging (MRI) is gold standard to evaluate CNS involvement in neurosarcoidosis, despite the reported high sensitivity but low specificity in the diagnosis. Case presentation: Here, we describe a 52-year-old man that presented to our hospital with a 10-year history of focal seizures, progressive cognitive decline and motor impairment. Neurological examination revealed ataxic gait, bilateral telekinetic and postural tremor, brisk reflexes, left extensor plantar response and hypoesthesia to the right side of body. Brain 3T-magnetic resonance imaging (MRI) showed a leukoencephalopathy with multifocal nodular lesions hyperintense on T2/ fluid attenuated inversion recovery (FLAIR) weighted images involving basal ganglia, periventricular and deep white matter. The interpretation of this pattern on conventional MRI was unclear, opening a challenge on the differential diagnosis between inflammatory, infective or neoplastic disorders. Thus, to better understand the nature of these nodules, single-voxel ^1^H-magnetic resonance spectroscopy (^1^H-MRS), contrast enhanced computed tomography (CT) scan and fluorine-18-fluorodeoxyglucose-positron emission tomography (^18^F-FDG-PET)/3T-MRI were performed. The parenchymal multifocal lesions exhibited slight *N*-acetyl-aspartate/creatine reduction without abnormal peaks on ^1^H-MRS, enhancement after the administration of contrast agent on CT and hypermetabolism on ^18^F-FDG-PET/3T-MRI. All these findings excluded primary neoplasms, metastasis, neurotuberculosis, neurocysticercosis and brain abscess, strongly suggesting a diagnosis of neurosarcoidosis. Therefore, a whole-body ^18^F-FDG-PET/CT was performed in order to identify subclinical extraneural sarcoidosis localizations, and a hypermetabolic nodule of the left lung upper lobe was found. Subsequently, a biopsy documented the presence of systemic sarcoidosis, supporting a diagnosis of probable neurosarcoidosis. Conclusions: This case demonstrated that a multimodal neuroimaging approach can provide different but complementary evidences to suspect sarcoidosis, especially in apparently CNS isolated forms.

## 1. Introduction

Neurosarcoidosis is a heterogeneous clinical condition exhibiting different types of lesions on imaging, as it can affect any part of the nervous system, such as the meninges, brain, spinal cord, cranial and peripheral nerves [1]. The diagnosis is a challenge when the disease is restricted to the central nervous system (CNS) in absence of involvement of other organs. Noncaseating sarcoid granulomas have to be differentiated from other disorders that may mimic neurosarcoidosis, such as infectious and inflammatory diseases or malignant neoplasms [1]. To the best of our knowledge, this is the first description of a neurosarcoidosis case in which conventional and advanced neuroimaging techniques were performed in order to investigate structural, biochemical and functional features of multifocal brain lesions and to drive the differential diagnosis. This report would be helpful in showing the usefulness of a combined radiological and nuclear medicine imaging in the diagnosis differential of nodular brain lesions. 

## 2. Case Report

**Figure 1:**
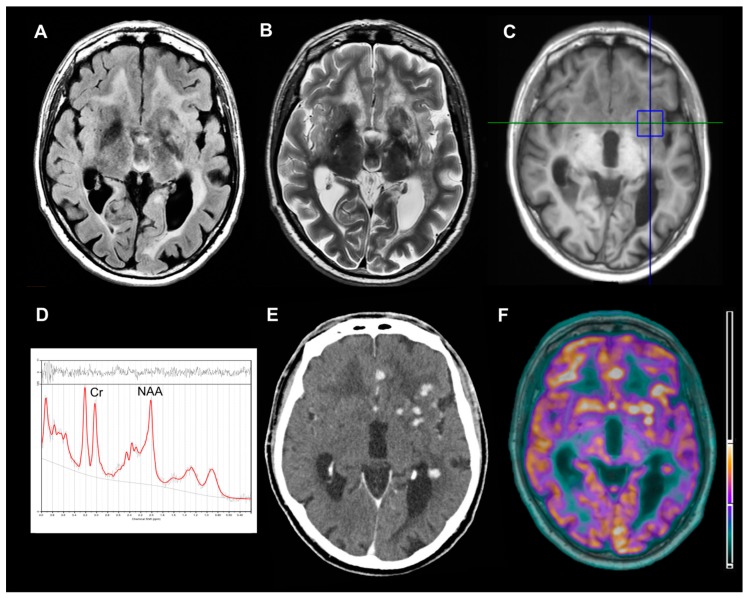
Structural, spectroscopic and metabolic findings of the brain nodular lesions. Axial fluid attenuated inversion recovery (FLAIR)-weighted (**A**) and T2-weighted (**B**) 3T-magnetic resonance imaging (MRI) images demonstrating several parenchymal nodular lesions and leukoencephalopathy. Single-voxel ^1^H-magnetic resonance spectroscopy (^1^H-MRS) involving basal ganglia lesions (**C**) and relative spectrum (**D**) showing *N*-acetyl-aspartate/creatine (NAA/Cr) reduction, without abnormal lactate or lipids peaks (NAA/Cr ratio was 0.873). Axial contrast-enhanced computed tomography (CT) (**E**) and fluorine-18-fluorodeoxyglucose-positron emission tomography (^18^F-FDG-PET)/3T-MRI (**F**) images demonstrating iopromide enhancement and hypermetabolism of all nodular lesions (the units are representative of percentage of overlap between MRI and PET).

**Figure 2:**
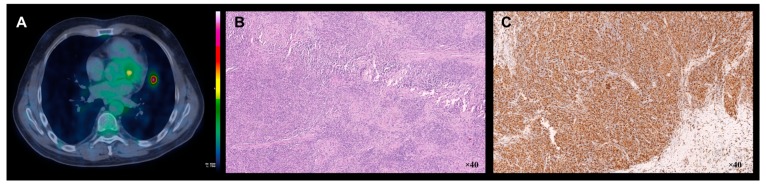
Metabolic and histopathological features of the lung granulomatous lesion. Axial chest ^18^F-FDG-PET/CT image (**A**) demonstrating increased metabolism in peribronchovascular nodule localized in the lingular segment of the left lung upper lobe (the units L are the intensity of PET activity, while units W are the intensity of overlap between PET and CT). Lung biopsy: haematoxylin–eosin stain and immunophenotyping showing non-necrotizing granulomatous inflammatory reaction (**B**) with accumulation of Cluster of Differentiation 68 positive (CD68+) macrophages (**C**).

A 52-year-old man presented to our hospital with a 10-year history of focal seizures, progressive cognitive decline and motor impairment. A written informed consent was obtained from the patient. Neurological examination revealed ataxic gait, bilateral telekinetic and postural tremor, brisk reflexes, left extensor plantar response and hypoesthesia to the right side of body. Cranial and peripheral nerves were intact, and no systemic manifestations were detectable. Laboratory results were not suggestive of any inflammatory or infectious disease. Cerebrospinal fluid (CSF) analysis indicated high levels of proteins and presence of oligoclonal bands. Chest imaging, including a high-resolution computed tomography (HRCT), resulted completely normal during previous years. Brain 3T-magnetic resonance imaging (MRI) showed a leukoencephalopathy with multifocal nodular lesions hyperintense on T2/fluid attenuated inversion recovery (FLAIR) weighted images involving basal ganglia, periventricular and deep white matter (Figure 1A,B). To better understand the nature of these nodules, single-voxel ^1^H-magnetic resonance spectroscopy (^1^H-MRS), contrast enhanced computed tomography (CT) scan and fluorine-18-fluorodeoxyglucose-positron emission tomography (^18^F-FDG-PET)/3T-MRI were performed. The parenchymal multifocal lesions exhibited slight *N*-acetyl-aspartate/creatine reduction without abnormal peaks on ^1^H-MRS (Figure 1C,D), enhancement after the administration of contrast agent (iopromide) on CT (Figure 1E) and hypermetabolism on ^18^F-FDG-PET/3T-MRI (Figure 1F). Furthermore, no leptomeningeal enhancement was detected. All these findings excluded primary neoplasms, metastasis, neurotuberculosis, neurocysticercosis and brain abscess, strongly suggesting a diagnosis of neurosarcoidosis [2]. Therefore, a whole-body ^18^F-FDG-PET/CT was performed in order to identify subclinical extraneural sarcoidosis localizations [1]. Then, a hypermetabolic peribronchovascular nodule of the left lung upper lobe was found (Figure 2A). Whole-body ^18^F-FDG-PET/CT was performed 65 minutes after the injection of 370 MBq of ^18^F-FDG by using three minutes/bed 2D scan. The patient was fasted for six hours. Brain ^18^F-FDG-PET/3T-MRI was acquired 45 minutes after 185 MBq of ^18^F-FDG injection. The patient was placed in a quiet room 20 minutes before injection and instructed to not to speak and read. The 10 minutes PET scan of the brain was corrected for movement by using a contemporary blood oxygenation level dependent (BOLD) sequence. Given that the patient has always refused brain sampling, pulmonary biopsy (Figure 2B,C) documented the presence of systemic sarcoidosis, supporting a diagnosis of probable neurosarcoidosis according to established diagnostic criteria [1]. 

Hence, the patient was promptly treated with prednisone 75 mg/day. Because of the lack of response after three months, methotrexate 12.5 mg/week was added, reducing prednisone’s posology at 50 mg/day. At three months follow-up, his motor impairment improved. Furthermore, seizures were controlled with carbamazepine 800 mg/day and phenobarbital 50 mg/day. 

## 3. Discussion 

Sarcoidosis of CNS without systemic manifestation can be present in approximately 10% to 19% of patients with neurosarcoidosis [1]. This condition might manifest as multiple or solitary nodules, periventricular white matter lesions, cranial nerves and meningeal thickening with contrast enhancement, hydrocephalus, involvement of hypothalamic–pituitary axis or long-extending myelitis. Brain parenchymal lesions may be seen in about 20% of cases [1]. MRI is the study of choice to evaluate CNS involvement but although has high sensitivity (82%–97%) it lacks in specificity [1]. Indeed, differentiating these nodular lesions from neoplastic (such as primary neoplasms and metastasis), or infective disorders (such as neurotuberculosis, neurocysticercosis and brain abscess), especially in absence of leptomeningeal involvement, may be challenging. For these reasons, advanced neuroimaging techniques may provide further support to the differential diagnosis. ^18^F-FDG-PET is very useful to identify CNS and extraneural localization of sarcoidosis [1] showing an increased metabolic uptake, despite discrepancy between MRI and FDG-PET findings that have been previously reported in cases of spinal and brain sarcoidosis [3,4,5]. In our case, no MRI and FDG-PET imaging discordance was found, and the hypermetabolic pattern of the multifocal brain lesions allowed us to exclude a diagnosis of neurocysticercosis and neurotuberculosis [2,6], in which lesions typically show a decreased metabolic uptake. However, an increased metabolic uptake may be detected not only in the neurosarcoidosis but also in metastasis, primary neoplasms and brain abscess. ^1^H-MRS allowed us to exclude these alternative diagnoses, because it showed a non-specific pattern of slight *N*-acetyl-aspartate/creatine reduction without abnormal peaks. Indeed, metastasis and primary neoplasms display suppressed *N*-acetyl-aspartate and creatine with elevated choline and lactate; whereas brain abscess are typically associated to abnormal peaks of lactate, lipids, amino acids, acetate and succinate [2,7]. 

Furthermore, cerebrospinal fluid abnormalities can be found in more than 50% of neurosarcoidosis, but none of them are specific: elevated CFS proteins and the presence of oligoclonal bands are seen in about 60% and 20% of patients respectively [1]. Indeed, the diagnosis of neurosarcoidosis is supported by the identification of granulomas in one or more organs and direct biopsy of neurologic tissue remains a key element to perform a diagnosis of definite neurosarcoidosis. Brain biopsy is not always easy to propose, and it is not free from side effects whereas the lung is the commonest site to sample [1]. Despite recent criteria clarification when considering the diagnosis [1], this lack of characteristic lesions and specific serologic or clinical findings could determine diagnostic challenges.

## 4. Conclusions

This case demonstrates that a multimodal neuroimaging approach [8] might provide different but complementary evidences to suspect sarcoidosis, especially in apparently CNS isolated forms. Thus, the use of radiological and nuclear medicine imaging should be considered in the diagnostic workup of brain nodular lesions.
